# Cultivar-Specific Changes in Primary and Secondary Metabolites in Pak Choi (*Brassica Rapa*, Chinensis Group) by Methyl Jasmonate

**DOI:** 10.3390/ijms18051004

**Published:** 2017-05-07

**Authors:** Moo Jung Kim, Yu-Chun Chiu, Na Kyung Kim, Hye Min Park, Choong Hwan Lee, John A. Juvik, Kang-Mo Ku

**Affiliations:** 1 Division of Plant and Soil Sciences, West Virginia University, Morgantown, WV 26506, USA; mjkim@mail.wvu.edu (M.J.K); yuchiu@mix.wvu.edu (Y.-C.C.); 2 Department of Bioscience and Biotechnology, Konkuk University, Seoul 143-701, Korea; god_1012@hanmail.net (N.K.K.); ramgee@naver.com (H.M.P.); chlee123@konkuk.ac.kr (C.H.L.); 3 Department of Crop Sciences, University of Illinois at Urbana-Champaign, Urbana, IL 61801, USA; juvik@illinois.edu

**Keywords:** glucosinolate, isothiocyanate, methyl jasmonate, pak choi, *Brassica rapa*

## Abstract

Glucosinolates, their hydrolysis products and primary metabolites were analyzed in five pak choi cultivars to determine the effect of methyl jasmonate (MeJA) on metabolite flux from primary metabolites to glucosinolates and their hydrolysis products. Among detected glucosinolates (total 14 glucosinolates; 9 aliphatic, 4 indole and 1 aromatic glucosinolates), indole glucosinolate concentrations (153–229%) and their hydrolysis products increased with MeJA treatment. Changes in the total isothiocyanates by MeJA were associated with epithiospecifier protein activity estimated as nitrile formation. Goitrin, a goitrogenic compound, significantly decreased by MeJA treatment in all cultivars. Changes in glucosinolates, especially aliphatic, significantly differed among cultivars. Primary metabolites including amino acids, organic acids and sugars also changed with MeJA treatment in a cultivar-specific manner. A decreased sugar level suggests that they might be a carbon source for secondary metabolite biosynthesis in MeJA-treated pak choi. The result of the present study suggests that MeJA can be an effective agent to elevate indole glucosinolates and their hydrolysis products and to reduce a goitrogenic compound in pak choi. The total glucosinolate concentration was the highest in “Chinese cabbage” in the control group (32.5 µmol/g DW), but indole glucosinolates increased the greatest in “Asian” when treated with MeJA.

## 1. Introduction

Pak choi (*Brassica rapa*, Chinensis group) is a cool-season crop similar to many other *Brassica* vegetables, such as kale and broccoli, and was domesticated in China [1]. Pak choi is a popularly-consumed vegetable in China and is showing an increase in consumption in Europe and North America, primarily due to its comparatively mild flavor [1,2]. In the U.S., its production is increasing for farmer’s markets and community-supported agriculture [1]. As a *Brassica* vegetable, pak choi provides a number of phytonutrients, in particular glucosinolates (19.36–63.43 µmol/g DW according to Wiesner et al. [3]). Although its popularity is increasing, the nutritional quality of pak choi has not been thoroughly investigated, and only a few studies have reported glucosinolate profiles from pak choi [3,4].

**Figure 1:**
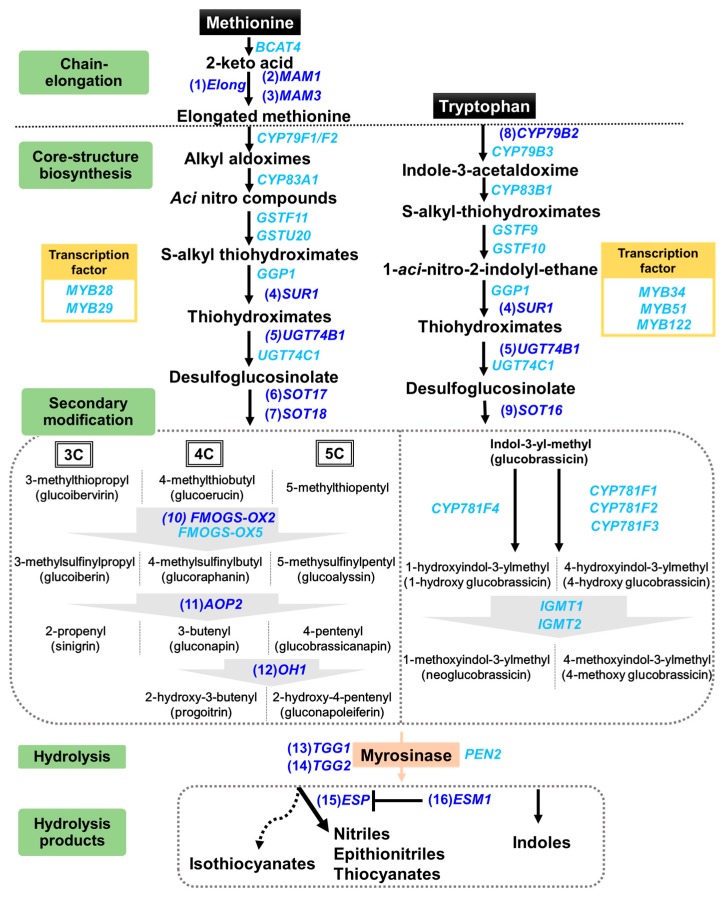
Schematic biosynthetic pathway of aliphatic and indole glucosinolates and involved genes (modified from Yi et al. [16]). Genes following numbers in brackets are analyzed in this study (Table S1). Gene names in light blue without a number were not analyzed in this study. The dashed line separates the biosynthesis pathway into three sections—chain elongation, core-structure biosynthesis, and secondary modification with addition of glucosinolate hydrolysis products. The dashed lines between compounds are to clarify and group the compounds from the same precursor or in the same group. Dotted arrow indicates another reaction pathway without involving of *ESP*. *ESM1* regulates *ESP* (bar and dash).

Pak choi contains a number of glucosinolates including gluconapin, glucobrassicanapin, progoitrin, glucobrassicin and neoglucobrassicin [3,4]. Glucosinolates are nitrogen- and sulfur-containing secondary metabolites derived from amino acids. Depending on their structure and precursor amino acid, glucosinolates are classified into three major groups: aliphatic (from methionine), indole (from tryptophan) and aromatic (from phenylalanine or tyrosine) glucosinolates (Figure 1) [5]. The first two steps in the biosynthetic pathway facilitate chain elongation and core structure formation, converting the precursor amino acid into desulfoglucosinolates after which final products are formed through secondary structural modifications [6]. Once formed and stored in the vacuoles of cells, glucosinolates can be hydrolyzed by an endogenous enzyme myrosinase following cellular disruption [7]. After glucosinolate hydrolysis, a few forms of hydrolysis products including isothiocyanates, nitriles, epithionitriles, thiocyanates and indoles are formed. The concentrations of specific hydrolysis products are determined by a number of factors including the activity of epithiospecifier protein (ESP) and epithiospecifier protein modifier 1, pH and the presence and concentration of certain metal ions (Figure 1). The potential health benefits and bioactivity of glucosinolates are attributed to their hydrolysis products, not the parent glucosinolates. A number of cell culture and pre-clinical studies have reported that *Brassica* vegetables and glucosinolate hydrolysis products are beneficial against carcinogenesis [8,9,10]. However, it was also shown that glucosinolate concentration was positively correlated with quinone reductase-inducing activity, a biomarker for anti-carcinogenic activity, in arugula (*Eruca sativa*) and horseradish (*Armoracia rusticana*) [11,12]. Therefore, increasing glucosinolate concentration could be a good strategy to enhance the potential health benefits of *Brassica* vegetables including pak choi.

Glucosinolates are a secondary metabolite whose biosynthesis can be induced by various biotic and abiotic factors [13]. Since glucosinolates are involved in plant defense, insect damage or physical wounding can induce glucosinolate biosynthesis. Glucosinolate concentrations can also be regulated by sulfur and nitrogen fertilization. In addition, growing degree days, solar radiation, number of days after transplanting and precipitation have been shown to affect glucosinolate biosynthesis [14]. These studies suggest that cultural practices that are effective and economic to apply can be developed to increase glucosinolate concentration and to improve the potential health benefits of glucosinolate-containing crops. It has been suggested that aliphatic, indole and aromatic glucosinolates biosynthesis have different regulatory mechanisms. Brown et al. [15] have reported that in broccoli, aliphatic glucosinolates are primarily controlled by genetic factors (accounting for >60% of the total variance), whereas indole glucosinolates are inducible compounds that are primarily influenced by the environment and cultural conditions or insect damage [14]. This report found that indole glucosinolates are relatively easier to increase by manipulating cultural conditions compared to aliphatic glucosinolates. In addition, conventional production systems minimize plant biotic stress by applying fungicides and insecticides, resulting in lower concentrations of indole glucosinolates in *Brassica* crops.

Previous studies have found that exogenous treatment of methyl jasmonate (MeJA) can induce indole glucosinolate biosynthesis in *Brassica* vegetables [14,17,18]. MeJA is registered in the Environmental Protection Agency (EPA) and is certified as a safe compound for all food commodities when applied preharvest [19]. Additionally, only 2–4 days are needed to elevate indole glucosinolate concentration prior to harvest in *Brassica oleracea* or *B*. *rapa*. [4,17] and, therefore, does not affect crop yield. Indole glucosinolates such as neoglucobrassicin are normally not accumulated at higher concentrations without severe herbivore or tissue damage, but can be regulated by MeJA. Ku et al. [18] have reported increased concentrations of glucobrassicin, an indole glucosinolate, by MeJA in the leaf tissues of the kale cultivars “Dwarf Blue Curled Vates” and “Red Winter” (98–166% of increase) in a two-year study while no significant change in aliphatic glucosinolate concentrations was observed. In the leaves of “Green Magic” and “VI-158” broccoli, indole glucosinolates also increased by MeJA treatment, whereas aliphatic glucosinolates were much less affected [17].

Although MeJA effects on pak choi glucosinolates have previously been reported [4,20], these studies only focused on major glucosinolates or analyzed one cultivar. Moreover, the profile of glucosinolate hydrolysis products, which are ultimately responsible for the bioactivity and potential health benefits, has not been investigated in pak choi. Considering its increasing popularity, pak choi merits further study on nutritional composition and the strategies to improve its health-promoting value. The objective of this study was to determine the metabolite changes in pak choi associated with foliar application of MeJA. Glucosinolates and their hydrolysis products, as well as the expression of genes in glucosinolate biosynthesis were analyzed in five pak choi cultivars to evaluate the effect of MeJA treatment on glucosinolate metabolism. Primary metabolites including amino acids, organic acids, sugars and sugar derivatives were analyzed to better understand how MeJA treatment changes metabolite flux from primary to secondary metabolites in pak choi.

## 2. Results and Discussions

### 2.1. Glucosinolate Concentrations

**Figure 2:**
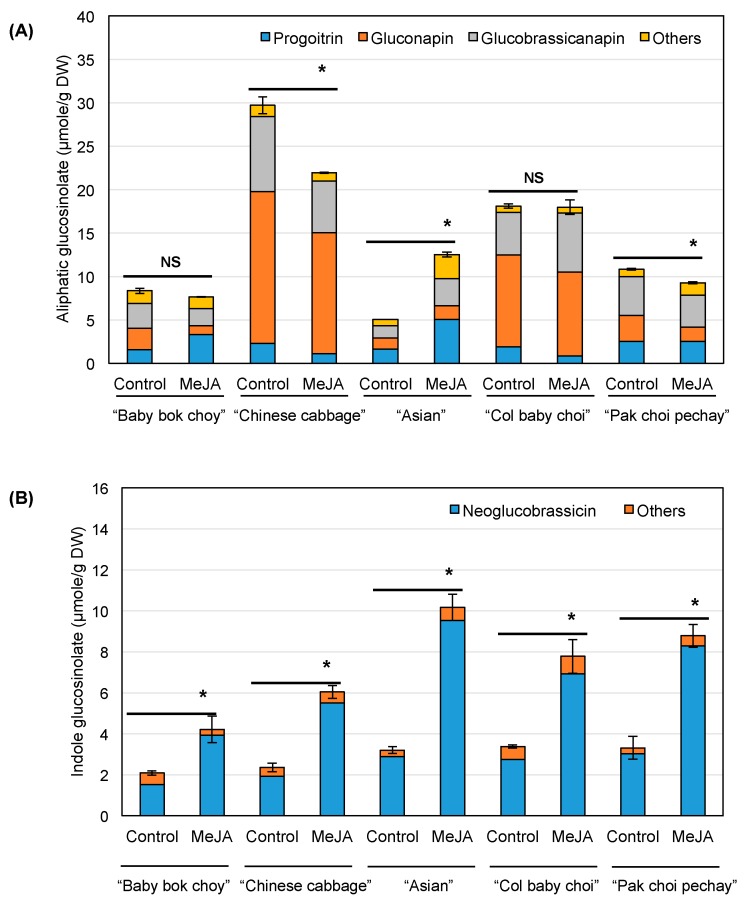
Aliphatic (**A**) and indole (**B**) glucosinolate concentration in control and MeJA-treated pak choi. Data are presented as the mean concentration ± the standard error of the total concentration (*n* = 3). Asterisks (*) above the bar indicate a significant difference of total concentration between treatments by Student’s *t*-test at *p* ≤ 0.05. MeJA, methyl jasmonate; DW, dry weight; NS, not-significant.

A total of 14 glucosinolates were detected with gluconapin and glucobrassicanapin as the predominant glucosinolates, representing 7–54% and 14–31% of the total glucosinolate concentration, respectively, depending on cultivar and treatment (Figure 2). Other aliphatic glucosinolates detected in pak choi included glucoiberin, progoitrin, glucoalyssin, gluconapoleiferin, glucoraphanin, sinigrin and glucoerucin (Table S2). The indole glucosinolates 4-hydroxyglucobrassicin, glucobrassicin, 4-methoxyglucobrassicin and neoglucobrassicin and aromatic glucosinolate gluconasturtiin were also present in the five pak choi cultivars. Our results are in agreement with a previous study that reported gluconapin and glucobrassicanapin as the major glucosinolates in pak choi [3]. Glucosinolate composition differed among cultivars and treatments. Gluconapin and glucobrassicanapin concentrations in the control ranged from 1.03 to 17.46 µmol/g DW and from 1.41 to 8.65 µmol/g DW, respectively (Figure 2). Sinigrin was only detected in control “Baby bok choy” (Supplementary Table S2). Glucoraphanin and glucoerucin were also detected in selected cultivars. These results agree with a previous report showing significant variation in glucosinolate concentrations among different pak choi cultivars [3,4]. Additionally, Wiesner et al. [3] reported that aliphatic glucosinolates represented 92–98% of the total glucosinolates, similar to our result (51–91% depending on cultivar and treatment). The control “Chinese cabbage” was found to contain the highest concentration of total glucosinolates, suggesting that this cultivar may possess the greatest glucosinolate-related health-promoting properties under standard production conditions among the five cultivars investigated in this study.

Glucosinolate composition changed in response to MeJA treatment differentially among cultivars (Figure 2). For example, gluconapin and glucobrassicanapin decreased in “Baby bok choy”, “Chinese cabbage” and “Pak choi pechay”, while increasing in ”Asian” with MeJA treatment. Similarly, progoitrin increased in ‘Baby bok choy’ and ‘Asian’, but decreased in the other cultivars when treated with MeJA. However, neoglucobrassicin, an indole glucosinolate, increased in all cultivars after MeJA treatment by 153–229%. Glucobrassicin also increased in “Asian”, “Col baby choi” and “Pak choi pechay”, while a decreasing 4-methoxyglucobrassicin concentration was found in “Baby bok choy” and “Chinese cabbage”. Our result is in agreement with the report by Zang et al. [4] who showed a significant increase in indole glucosinolate concentrations with MeJA treatment in four pak choi cultivars. However, they found increased aliphatic glucosinolates in only one cultivar, indicating that aliphatic glucosinolate concentrations are less affected by MeJA compared to indole glucosinolates, and their change could be cultivar specific, consistent with our result. MeJA’s effect on glucosinolates has been reported in other *Brassica* vegetables, including broccoli, kale, cauliflower and Chinese cabbage [17,18,21,22]. These studies reported that indole glucosinolates, in particular neoglucobrassicin, increased while aliphatic glucosinolates were less influenced by MeJA. However, we found that aliphatic glucosinolates can also significantly change in response to MeJA in pak choi, with variation in their change depending on compound and cultivar, indicating that glucosinolate changes by MeJA treatment differ among species, crops and cultivars. When treated with MeJA, “Asian” showed the greatest increase in the total glucosinolate concentration (2.7-fold), indicating that this cultivar has the greatest sensitivity to MeJA treatment among the investigated cultivars, and thus, MeJA can be an effective agent to elevate glucosinolates of this pak choi cultivar. Additionally, changes in glucosinolate concentrations were partially associated with differential expression levels of genes involved in glucosinolate biosynthesis in response to MeJA treatment, depending on cultivar. In particular, the expression level of *OH1*, a gene converting gluconapin to progoitrin (Figure 1), increased by 14.4-, 49.8- and 7.3-fold in “Baby bok choy”, “Asian” and “Pak choi pechay”, respectively (Supplementary Table S1). The expression level of *OH1* was positively correlated with progoitrin (*r* = 0.902, *p* = 0.0004, *n* = 10). Gluconapoleiferin, where the same genes are responsible for its biosynthesis, increased in “Baby bok choy”, “Asian” and “Pak choi pechay” with upregulation of those genes (Table S1). The expression level of *OH1* was also positively correlated with gluconapoleiferin (*r* = 0.846, *p* = 0.0021, *n* = 10). Although indole glucosinolates, in particular neoglucobrassicin, were more affected by MeJA than aliphatic glucosinolates, changes in indole glucosinolate concentration and related gene expression have been reported [17,20]. However, how aliphatic glucosinolates are affected by MeJA has been less elucidated.

### 2.2. Glucosinolate Hydrolysis Products

We detected 14 hydrolysis products including isothiocyanates, nitriles, indoles and oxazolidine-thione (as shown by Rask et al. [23]) and their changing concentrations related to MeJA treatment depended on cultivar, similar to the glucosinolates (Table 1). The 3-butenyl isothiocyanate, 4-pentenyl isothiocyanate and phenethyl isothiocyanate, the hydrolysis product of gluconapin, glucobrassicanapin and gluconasturtiin, respectively, decreased with MeJA treatment in all cultivars, except for increased 4-pentenyl isothiocyanate and phenethyl isothiocyanate in “Asian”. However, nitriles, including crambene (from progoitrin), 1-cyano-3,4-epithiobutane (from gluconapin), 1-cyano-4,5-epithiopentane (from glucobrassicanapin), 1-cyano-2-hydroxy-3,4-epithiobutane (from progoitrin) and an oxazolidine-thione goitrin (from progoitrin), changed differentially among cultivars with MeJA treatment. Most nitriles increased in “Asian”but goitrin decreased in all cultivars with MeJA treatment. Crambene increased in “Baby bok choy” and “Asian”, but decreased in “Chinese cabbage” and “Col baby choi”. In contrast to isothiocyanates and nitriles, 1-methoxyindole-3-carbinol, a hydrolysis product of neoglucobrassicin, and other compounds, including 1-methoxyindole-3-carboxaldehyde, 1-methoxyindole-3-acetonitrile and indole-3-acetonitrile, significantly increased in all five cultivars, except for no statistical change in 1-methoxyindole-3-carbinol in “Chinese cabbage”.

Although the glucosinolate profile and hydrolysis products from indole glucosinolates have been investigated [3], a full profile of glucosinolate hydrolysis products has not been reported for pak choi. Ku et al. [18] observed differential hydrolysis product composition between “Dwarf Blue Curled Vates” and “Red Winter” kale and a significant increase in 1-methoxyindole-3-carbinol in both cultivars with MeJA treatment. Jasmonic acid also increased 1-methoxyindole-3-carbinol in “VI-158” and “Green Magic” broccoli, but depending on the concentration of MeJA [17]. Glucosinolate hydrolysis product concentrations clearly change with MeJA treatment, in particular the hydrolysis products of neoglucobrassicin.

**Figure 3:**
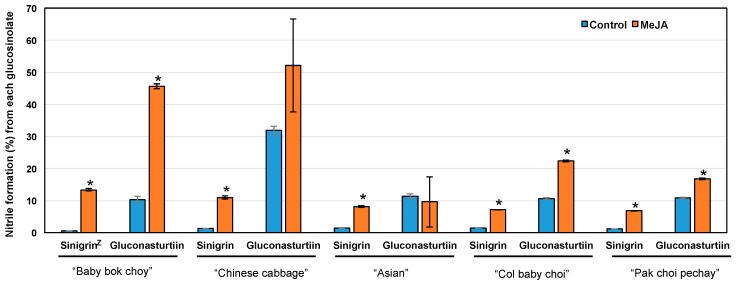
Nitrile formation (%) of control and MeJA-treated pak choi. Nitrile formation (%) is shown as the relative ratio of nitrile to the total hydrolysis product formed (sum of isothiocyanates and nitriles). Data are presented as the mean concentration ± standard error (*n* = 3). Asterisks (*) above the error bar indicate a significant difference between treatments within same glucosinolates substrate by Student’s *t*-test at *p* ≤ 0.05. ^z^ Substrate glucosinolate used in the estimation of nitrile formation activity [nitrile percentage (%) out of total hydrolysis products].

In addition to an increased amount of the hydrolysis product of neoglucobrassicin, we also found that hydrolysis products from aliphatic glucosinolates changed with MeJA treatment. For instance, 3-butenyl and 4-pentenyl isothiocyanates decreased by MeJA in all cultivars except for increased 4-pentenyl isothiocyanate in “Asian” (Table 1). This is probably related to the activity of ESP, which enhances the formation of epithionitriles over isothiocyanates [24]. We indirectly measured the ESP activity by incubating pak choi protein extract with the extract of horseradish, which has a simple glucosinolate profile [11], and found that nitrile formation (%) increased in all cultivars when treated with MeJA except for “Chinese cabbage” and “Asian” for the hydrolysis of gluconasturtiin (Figure 3). Although nitrile formation (%) of MeJA-treated “Asian” was significantly higher than control according to allyl isothiocyanate (from sinigrin), there was no significant difference in phenethyl isothiocyanate (from gluconasturtiin) (Figure 3). There was a significant correlation (*r* = 0.700, *p* = 0.0239, *n* = 10) between nitrile formation from sinigrin and nitrile formation from gluconasturtiin. Additionally, we found a significant correlation (*r* = 0.925, *p* < 0.0001, *n* = 10) between nitrile formation from sinigrin and nitrile formation from neoglucobrassicin, where the nitrile formation (%) from neoglucobrassicin was determined as the percentage of 1-metoxyindole-3-acetonitrile to the total hydrolysis products (Table 1). Other compounds, such as crambene, 1-cyano-3,4-epithiobutane, 1-cyano-4,5-epithiopentane and 1-cyano-2-hydroxy-3,4-epithiobutane, also changed with MeJA treatment depending on cultivar. These results indicate that ESP activity in general increased with MeJA in most pak choi cultivars and thus partially explains why isothiocyanates tended to decrease with MeJA application. We also found that MeJA treatment significantly reduced myrosinase activity in “Baby bok choy”, “Chinese cabbage” and “Pak choi pechay” cultivars, indicating a cultivar-specific response to MeJA (Supplementary Figure S1). Regardless of cultivar, goitrin, an oxazolidine-thione from progoitrin, decreased by MeJA. This is significant because goitrin is a goitrogenic compound that can disrupt hormone production in the thyroid gland by inhibiting uptake of iodine [25], and therefore, high level of goitrin intake could be a problem, especially under iodine malnutrition.

Hydrolysis products of glucosinolates have been considered to reduce the risk of degenerative diseases, especially against carcinogenesis. The 3-butenyl isothiocyanate, a hydrolysis product of gluconapin, has shown anti-proliferative activity against various cancer cell lines including human prostate, lung, cervical, liver and breast cancers, as well as human neuroblastoma and osteosarcoma cell lines [8]. In particular, prostate cancer PC-3 cells had the greatest inhibition by 3-butenyl isothiocyanate with IC_50_ (50% inhibitory concentration) and IC_70_ values of 0.041 and 0.060 μg/mL, respectively, compared to the positive control camptothecin (IC_50_ of 121.60 μM; 42.36 μg/mL). Isothiocyanates have been generally accepted to possess a greater bioactivity compared to other forms of glucosinolate hydrolysis products, but some compounds in other forms have also been studied for their potential health benefits. Crambene, a nitrile from progoitrin, was reported to increase mRNA expression of quinone reductase, as well as the activity of quinone reductase and glutathione-*S*-transferase [26,27] in mouse hepatoma Hepa1c1c7 cells (0.1–10 mM) or in adult male CDF 344 rats (fed 50 mg/kg of crambene for seven days), indicating a potential anticarcinogenic property of crambene. Another compound, 1-methoxyindole-3-carbinol, a hydrolysis product of neoglucobrassicin, inhibited the growth of human colon cancer DLD-1 and HCT-116 cells in a dose-dependent manner at 10–100 μM [9]. However, the effect of 1-methoxyondole-3-carbinol on carcinogenesis is inconclusive. When applied to murine hepatoma Hepa1c1c7 cells at 50 μM or administered to Winster rat at 570 μmol/kg body weight, 1-methoxyindole-3-carbinol significantly increased cytochrome P-450 1A1, indicating that this compound may help carcinogenesis [28]. Another study reported that male transgenic C57BL/6-Tg(TRAMP)8247Ng/J mouse (transgenic adenocarcinoma of mouse prostate) fed a diet containing 10% of indole glucosinolate-elevated broccoli powder showed no difference from the mouse fed a diet containing normal broccoli powder in the reduction of prostate carcinogenesis [29]. Although not analyzed in this study, indole-3-carbinol, generated from glucobrassicin, has been reported for its potential anticarcinogenic activity in cell culture and preclinical studies, and it is sometimes reported that 1-methoxyindole-3-carbinol may possess a greater bioactivity compared to indole-3-carbinol [9,30,31,32]. It was suggested that methylation increases hydrophobicity of the compound and enhances cell membrane penetration, and therefore, 1-methoxyindole-3-carbinol may have a greater bioactivity [17]. The concentration of 1-methoxyindole-3-acetonitrile was significantly increased by MeJA treatment due to the upregulation of neoglucobrassicin synthesis and high ESP activity (indirectly estimated as nitrile formation), but there was no report on a health-promoting effect of this compound; therefore, further studies are needed.

This study revealed that MeJA not only increases total glucosinolates, but also changes hydrolysis product concentrations. Isothiocyanates were in general reduced, but 1-methoxyindole-3-acetonitrile increased in all five cultivars, and 1-methoxyindole-3-carbinol increased in four cultivars. Goitrin, a goitrogenic compound, decreased in all cultivars. In particular, “Asian” had increased 4-pentenyl isothiocyanate, crambene and 1-methoxyindole-3-carbinol, but decreased goitrin by MeJA. Our results suggest that MeJA can significantly change glucosinolate metabolites, and “Asian” is specifically responsive to MeJA. Based on the metabolite changes in “Asian” by MeJA, “Asian” might be an excellent choice to improve the health-promoting values of pak choi using MeJA.

### 2.3. Primary Metabolites

It has been suggested that exogenous MeJA can alter primary metabolites, such as sugars, organic acids and amino acids, and these changes may affect glucosinolate biosynthesis [33]. Therefore, analysis of these metabolites may help to understand their further transition to secondary metabolites. Although the MeJA effect on glucosinolate has been studied in a few *Brassica* crops, how MeJA affects primary metabolism has not been well reported. Primary metabolites with variable importance in projection (VIP) scores over 1.0 were selected based on the cut-off value for VIP advocated by Wold [34] to separate terms that do not make an important contribution to the dimensionality reduction involved in PLS (partial least squares) (VIP < 0.8) from those that might (VIP ≥ 0.8) (Table 2). A higher VIP score indicates a greater difference between treatments and, therefore, can be useful in selecting a biomarker that differs between treatments. According to the VIP scores, three amino acids (alanine, valine, glutamic acid), two organic acids (citric acid and cinnamic acid) and five sugars and sugar derivatives (glycerol, fructose, *myo*-inositol, galactose and maltose) among detected compounds were the most important primary metabolites that differentiate MeJA-treated pak choi from control plants (Table 2). A high VIP score of these compounds indicates that these compounds are more important biomarkers that describe the variation in the primary metabolites of pak choi.

**Figure 4:**
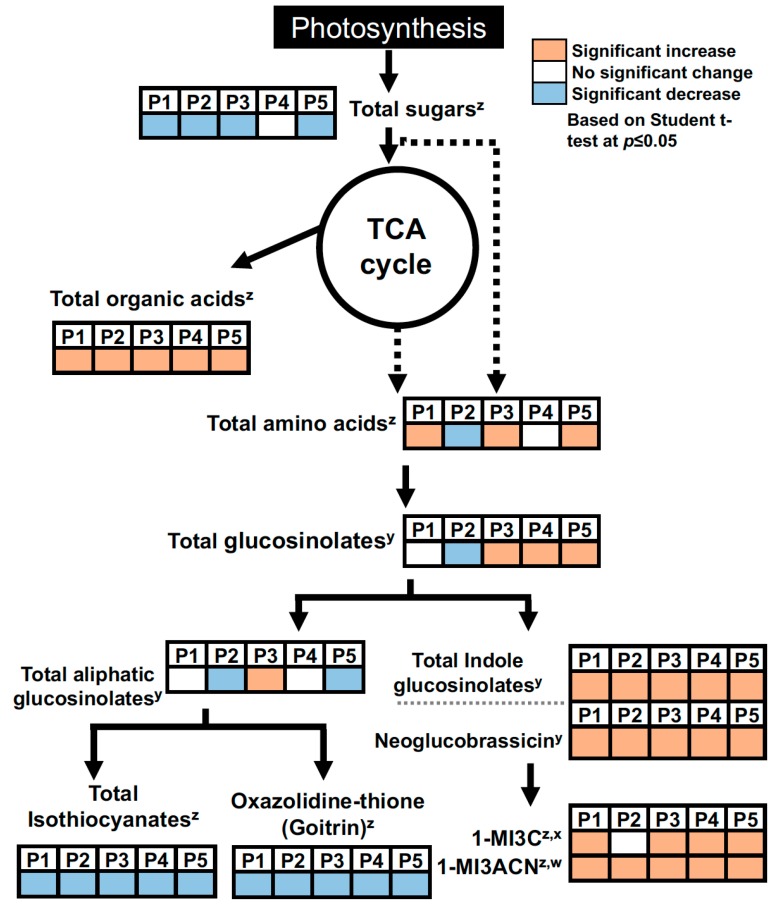
Summary of MeJA effect on primary and secondary metabolites. All data were compared to control by Student’s *t*-test at *p* ≤ 0.05. GS, glucosinolate; 1-MI3C, 1-methoxyindole-3-carbinol. P1, “Baby bok choy”; P2, “Chinese cabbage”; P3, ”Asian“; P4, “Col baby choi”; P5, “Pak choi pechay”; ^x^ 1-methoxyindole-3-carbinol; ^y^ based on compound concentration; ^w^ 1-methoxyindole-3-acetonitrile; ^z^ Based on peak intensity. The dotted line indicates the conversion is simplified biosynthesis without intermediates.

Individual metabolites varied with MeJA treatment among cultivars (Table 2 and Table S3). Among the metabolites with VIP score >1.0, levels of the amino acids alanine, valine and glutamic acid were significantly higher when treated with MeJA in “Pak choi pechay” (Table 2). In contrast, glutamic acid decreased in “Chinese cabbage” and “Col baby choi”. Organic acids also varied with MeJA treatment depending on cultivar. Citric acid increased in all cultivars with MeJA treatment. Cultivar-dependent changes with MeJA were also observed for sugar levels. MeJA treatment reduced fructose, maltose and galactose in “Asian”, but fructose increased in “Col baby choi”. Glycerol and *myo*-inositol increased in four cultivars. Among the selected metabolites, we found a general increase or no change in alanine and valine, increases in organic acids, decreases in mono- and di-saccharide sugars and elevated sugar alcohols. Although we observed increases in some amino acids depending on cultivar, Tytgat et al. [35] reported decreased amino acids in jasmonic acid-treated *B*. *oleracea* plants. They also reported reduced sugar concentration with jasmonic acid treatment, similar to our observations. Kim [33] reported reduced levels of hexose sugars and TCA cycle intermediates in MeJA-treated “Green Magic” broccoli leaves. These results and our data indicate that exogenous MeJA can decrease sugar levels and changes in secondary metabolism might partially be attributed to reduction of sugars, as sugars may provide the carbon skeleton for secondary metabolite biosynthesis (Figure 4). Since MeJA is synthesized from linolenic acid, MeJA may also affect fatty acid metabolism. When treated with MeJA, linolenic acid concentration, as well as fatty acid composition changed in mature green tomatoes [36]. When treated with MeJA, linolenic acid increased, while linoleic acid was reduced, suggesting a possible MeJA effect on fatty acids and their derivatives. In addition, changes in sugar metabolism could also have affected sugar alcohol biosynthesis, such as glycerol and *myo*-inositol.

To our knowledge, changes in primary metabolites in response to MeJA treatment have not been reported in pak choi. Additionally, information of primary metabolite changes by MeJA treatment in other *Brassica* crops is also lacking. Liang et al. [37] applied MeJA to turnip (*B*. *rapa* var. *rapa*) and found that most sugars and amino acids analyzed using nuclear magnetic resonance spectroscopy were reduced by MeJA, in contrast to our results. This difference from our result indicates that MeJA might affect primary metabolism differentially among crops. Moreover, our result shows that the MeJA effect varied among the five pak choi cultivars.

Many of the primary metabolites analyzed in this study play an important role in human diets. For instance, amino acids, in general, are involved in various biochemical mechanisms, such as protein synthesis, cell signaling, osmoregulation and metabolic regulation [38] with some amino acids also associated with mammalian immune systems [39]. Moreover, hexose sugars and organic acids are involved in the primary metabolisms such as the TCA cycle and are used as a precursor of amino acids and secondary metabolites (Figure 4). Therefore, understanding how these primary metabolites change by MeJA treatment in addition to secondary metabolites are important to improve the nutritional value of foods and for developing cultivation regimes to enhance the nutritional properties.

## 3. Materials and Methods

### 3.1. Plant Materials

The pak choi cultivars used in this experiment were “Baby bok choy” (Lake Valley Inc., Boulder, CO, USA), “Chinese cabbage” (Heirloom; Lake Valley Inc., Boulder, CO, USA), “Asian” (Livingston Seed, Columbus, OH, USA), “Col baby choi” (Burpee Seeds, Warminster, PA, USA) and “Pak choi pechay” (Burpee Seeds, Warminster, PA, USA). Seeds of each pak choi cultivar were germinated in a 32-cell plug tray filled with Sunshine LC1 professional soil mix (Sun Gro Horticulture, Vancouver, BC, Canada). Plants were grown in a greenhouse at the University of Illinois at Urbana-Champaign under a 25/18 °C and 14/10 h: day/night temperature and light regimes with additional HID (high-intensity discharge) lighting provided for 14 h (from 06:00 to 20:00). Four weeks after germination, plants in the vegetative growth stage were transplanted to a 1-L pot in the greenhouse and grown under the same environmental conditions. After four weeks, all aerial parts of the pak choi cultivars were sprayed with 500 µM MeJA in 0.1% ethanol. Control plants were applied with 0.1% ethanol. Then, nine pak choi plants were harvested for each treatment with three plants in each of three biological replicates, two days after spray. All of the samples were freeze-dried, ground to a fine powder and stored at <−20 °C prior to extraction.

### 3.2. Analysis of Glucosinolates

Extraction and quantification of glucosinolates using HPLC were performed following a previously published protocol [18]. Freeze-dried pak choi powder (0.2 g) and 2 mL of 70% methanol were added to a 10-mL tube (Nalgene, Rochester, NY, USA) and heated on a heating block at 95 °C for 10 min. After cooling on ice, 0.5 mL of glucosinalbin (1 mM; purified from seeds of *Sinapis alba*; and the concentration was confirmed using sinigrin standard (Sigma-Aldrich, St. Louis, MO, USA)) were added as an internal standard, and the mixture was centrifuged at 8000× *g* for 5 min at 4 °C. The supernatant was collected, and the pellet was re-extracted with 2 mL of 70% methanol at 95 °C for 10 min. A subsample (1 mL) from each pooled extract was transferred to a 2-mL microcentrifuge tube (Fisher Scientific, Waltham, MA, USA). A mixture of 1 M lead acetate and 1 M barium acetate (1:1, *v*/*v*) (0.15 mL) was added to precipitate protein. After centrifugation at 12,000× *g* for 1 min, each sample was loaded onto a column containing DEAE Sephadex A-25 resin that was pre-charged with 1 M NaOH and 1 M pyridine acetate (GE Healthcare, Piscataway, NJ, USA). Samples were incubated for 18 h with *Helix pomatia* Type-1 arylsulfatase (Sigma-Aldrich, St. Louis, MO, USA) for desulfation, and the desulfo-glucosinolates were eluted with 3 mL of Millipore-filtered deionized distilled water. Samples (100 µL) were injected to a high performance liquid chromatography (Agilent 1100 HPLC system, Agilent Technologies, Santa Clara, CA, USA) equipped with a binary pump (G1311A, Agilent Technologies, St. Clara, CA, USA), a vacuum degasser (G1322A, Agilent Technologies), a thermostatic column compartment (G1316A, Agilent Technologies), a diode array detector (G1315B, Agilent Technologies) and an autosampler (HP 1100 series G1313A, Agilent Technologies, Santa Clara, CA). An all-guard cartridge pre-column (Alltech, Lexington, KY, USA) and a Kromasil RP-C18 column (250 mm × 4.6 mm, 5-µm particle size, Supelco, Bellefonte, PA, USA) were used for glucosinolate separation. The flow rate was 1 mL/min with mobile Phase A (1 mM ammonium acetate containing 1% acetonitrile (*v*/*v*)) and B (100% acetonitrile), with the following elution profile: 0 min 0% B, 7 min 4% B, 20 min 20% B, 35 min 25% B, 36 min 80% B, 40 min 80% B, 41 min 0% B, and 50 min 0% B. The glucosinolates were detected at 229 nm. The UV response factor for each glucosinolate was used for quantification (Clarke, 2010). The identification of desulfo-glucosinolate profiles was validated using LC-tandem mass spectrometer (MS) (32 Q-Tof Ultima spectrometer, Waters Corp., Milford, MA, USA) coupled to HPLC (1525 HPLC system, Waters Corp.). The molecular ion and fragmentation patterns of individual desulfo-glucosinolates were compared to a previously published report [40].

### 3.3. Quantification of Glucosinolate Hydrolysis Products

Freeze-dried pak choi powder (50 mg) was suspended in 1 mL distilled water in a 2-mL microcentrifuge tube (Fisher Scientific, Waltham, MA, USA). Hydrolysis products were generated naturally by endogenous myrosinase in the absence of light at room temperature for 24 h. After adding 1 mL of dichloromethane, the samples were centrifuged at 12,000× *g* for 2 min, and the lower organic layer was collected. A gas chromatograph (GC) (6890N, Agilent Technologies) coupled to a MS detector (5975B, Agilent Technologies) equipped with an auto sampler (7683B, Agilent Technologies) and a capillary column (30 m × 0.32 mm × 0.25 µm J&W HP-5, Agilent Technologies) was used to determine glucosinolate hydrolysis products. A 1-μL sample of the dichloromethane extract was injected to the GC-MS with the split ratio of 1:1. After an initial temperature held at 40 °C for 2 min, the oven temperature was increased to 260 °C at 10 °C/min and held for 10 min [41]. Injector and detector temperatures were set at 200 and 280 °C, respectively. The flow rate of the helium carrier gas was set at 1.1 mL/min. Peaks were identified using standard compounds (goitrin, crambene, indole-3-acetonitrile and 3-butenyl isothiocyanate) or by comparing with the National Institute of Standards and Technology (NIST) library or previous publications [42,43] (Table S4).

### 3.4. Measurement of Nitrile Formation and Myrosinase Activity

Nitrile formation (%) was measured to estimate the ESP activity as ESP enhances the formation of nitriles over isothiocyanates. The nitrile formation of each pak choi cultivar was determined by incubating concentrated horseradish root extract with protein extract of pak choi. The horseradish extract was used as an exogenous substrate source of sinigrin and gluconasturtiin at the saturated level in order to minimize the reaction of pak choi protein with endogenous glucosinolates substrate. Subsequently, only hydrolysis products from sinigrin and gluconasturtiin were dominant compounds detected from GC-MS (Figure S2).

To measure the nitrile formation and myrosinase activity, freeze-dried pak choi powder (75 mg) was mixed with 1.5 mL of concentrated “1091” horseradish root extract [11] in 2-mL microcentrifuge tubes (10 g of horseradish were mixed with 100 mL of 70% methanol. This solution was centrifuged at 4000× *g* for 5 min. The supernatant of horseradish root extract was transferred to a beaker and boiled until all solvent was evaporated, then reconstituted with 50 mL of deionized water). The sinigrin and gluconasturtiin concentrations were 74 and 7 µmol/g DW, respectively. After centrifugation at 12,000× *g* for 2 min, 0.6 mL supernatant were transferred to a 1.5-mL Teflon centrifuge tube (Savillex Corporation, Eden Prairie, MN, USA), and then, 0.6 mL of dichloromethane were added. The tubes were placed upside down to minimize the loss of volatile compounds at room temperature for an hour. Then, tubes were vortexed and centrifuged at 12,000× *g* for 2 min. The dichloromethane organic layer was injected to GC-MS (Trace 1310 GC, Thermo Fisher Scientific, Waltham, MA, USA) coupled to a MS detector system (ISQ QD, Thermo Fisher Scientific, Waltham, MA, USA) and an auto sampler (Triplus RSH, Thermo Fisher ScientificA capillary column (DB-5MS, Agilent Technologies; 30 m × 0.25 mm × 0.25 µm capillary column) was used to determine glucosinolate hydrolysis products. After an initial temperature held at 40 °C for 2 min, the oven temperature was increased to 320 °C at 15 °C/min and held for 4 min. Injector and detector temperatures were set at 270 °C and 275 °C, respectively. The flow rate of the helium carrier gas was set at 1.2 mL/min. Standard curves of allyl isothiocyanate, 2-phenthyl isothiocyanate and 3-phenylpropionitrile (Sigma-Aldrich) were used for quantification, and the relative ratio of nitrile to the total hydrolysis products was calculated to determine ESP activity. The standard curve from allyl isothiocyanate was applied to quantify of 1-cyano-2,3-epithiopropane. Myrosinase activity was estimated as the total amount of hydrolysis products produced within 60 min [41]. One unit was defined as 1 μmole of the above four hydrolysis products released per min.

### 3.5. RNA Extraction and Quantitative Real-Time-PCR

Total RNA was isolated from control and MeJA-treated pak choi plants using RNeasy Mini Kit (QIAGEN, Hilden, Germany) following the manufacturer’s instructions. The quantity of RNA was measured using a NanoDrop 3300 spectrophotometer (Thermo Scientific, Waltham, MA, USA). One microgram of RNA was reverse-transcribed with Superscript™ III First-Strand Synthesis SuperMix for qRT-PCR (Invitrogen, Carlsbad, CA, USA) according to the manufacturer’s instructions. The qRT-PCR was carried out with the Power SYBR^®^ Green RT-PCR Master Mix (QIAGEN) using an ABI 7900HT Fast Real-Time PCR System (Applied Biosystems, Foster City, CA, USA) following the manufacturer’s instructions. The resulting cDNA samples were diluted to 1/10 (*v*/*v*) for qRT-PCR. The primer sets of glucosinolate biosynthesis genes, hydrolysis genes and transcription factor genes were designed based on the sequences published in the online database (Available online: http://www.ocri-genomics.org/bolbase/index.html) [6]. The primers were synthesized by Integrated DNA Technologies (Coralville, IA, USA). A final list of the primers used, the gene models from which they were created and classifications of the genes can be found in Supplementary Table S5. The results were expressed after normalization to the *Brassica rapa* actin gene (*BrACT1*) [3,17]. The relative expression ratio was determined with the equation 2^−∆∆*C*t^ using the *BrACT1* normalized ∆*C*t values generated by the ABI 7900HT Sequence Detection System Software 2.4 (Applied Biosystems).

### 3.6. Primary Metabolites Analysis by GC-Time-of-Flight-MS

Primary metabolites were analyzed following the method of Ku et al. [44] with minor modifications. Freeze-dried powder of pak choi (400 mg) was extracted with 10 mL of a mixture of methanol, deionized distilled water and chloroform (2.5:1:1, *v*/*v*/*v*) for 24 h using a Twist Shaker (Biofree, Seoul, Korea). The resulting mixture was centrifuged at 5000 rpm for 5 min (Universal 320, Hettich Zentrifugen, Tuttlingen, Germany). The supernatant of 900 μL was transferred to a 1.5-mL microcentrifuge tube. After adding of 400 μL distilled water and centrifugation at 5000 rpm for 5 min, 400 μL of the polar phase were transferred to another 1.5-mL tube and then concentrated using Modulspin 31 speed-vacuum concentrator (Biotron, Seoul, Korea). For the GC-MS analysis, the dried extract was oximated with 50 μL methoxyamine hydrochloride in pyridine (20 mg/mL) for 90 min at 30 °C using a thermomixer (Eppendoft, Hamburg, Germany), followed by silylation with 50 μL of *N*-methyl-*N*-(trimethylsilyl)-trifluoroacetamide (MSTFA) (Sigma-Aldrich) for 30 min at 37 °C using a thermomixer. The final concentration of the samples was 2.5 mg/mL for GC-time-of-flight (TOF)-MS analysis. The samples were then filtered through a 0.2-µm PTFE filter. Each biological replication (*n* = 3) from each pak choi cultivar sample (1 μL) was injected in triplicate with the split ratio of 1:5.

A GC system (7890A, Agilent Technologies), equipped with an autosampler (7693, Agilent Technologies) and a Pegasus HT TOF-MS (Leco Corporation, St. Joseph, MI, USA), and a capillary column (HP-5MS, 30 m × 0.25 mm × 0.25 µm, Agilent Technologies) were used for analysis. Chromatographic-grade helium with a constant flow of 1.0 mL/min was used as the carrier gas. The injector and transfer line temperatures were both set at 240 °C. The oven temperature was initially held at 75 °C for 2 min, then ramped to 300 °C at a rate of 15 °C/min and maintained at 300 °C for 3 min. The mass data collected in the electron ionization mode with 70 eV of ionization energy were used to conduct a mass scan ranges at *m*/*z* 50–1000. The average value from three analytical replications for each biological replication was used for statistical analysis. Identification of compounds was done by comparing with standard compounds or the NIST library (Table S3).

### 3.7. Statistical Analysis

All analyses were done with three biological replications (three plants per replication). Univariate analysis of variance (ANOVA) and Student’s *t*-test were performed using JMP Pro 12 (SAS Institute, Cary, NC, USA) to determine the MeJA effect on primary and secondary metabolites and gene expression changes. For primary metabolite data, raw data files were converted to CDF format (*.cdf) using the Leco ChromaTOF software program (Version 4.44, Leco Corp, Warrendale, PA, USA). After conversion, the MS data were processed using the metAlign software package (Available online: http://www.metalign.nl) to obtain a data matrix containing retention times, accurate masses and normalized peak intensities. The resulting data were exported to Excel (Microsoft, Redmond, WA, USA) for further analysis using MetaboAnalyst 3.0 (Available online: http://www.metaboanalyst.ca/faces/home.xhtml).

## 4. Conclusions

In the present study, the effects of foliar application of MeJA on glucosinolates, their hydrolysis products and primary metabolites were analyzed in five pak choi cultivars. The MeJA treatment significantly changed primary and secondary metabolite composition with the response to MeJA treatment differing among cultivars. In general, indole glucosinolates and their hydrolysis products were significantly increased by MeJA treatment, whereas other glucosinolates, in particular aliphatic glucosinolates, changed differentially depending on cultivar. Moreover, MeJA reduced goitrin, a goitrogenic compound produced from an aliphatic glucosinolate progoitrin, in all five cultivars. Amino acids, organic acids and sugar alcohols tended to increase, but mono- and di-saccharide sugars decreased with MeJA treatment, suggesting that the sugars may be a carbon source for secondary metabolite biosynthesis induced by MeJA treatment (Figure 4). Some gene expression data supported glucosinolate changes, for instance *OH1* for progoitrin biosynthesis. In conclusion, the results of this study suggest that MeJA treatment can act as an agent to regulate the primary and secondary metabolites in pak choi, and cultivar-specific responses to MeJA were found among the five pak choi cultivars. Additionally, these results can be used in developing cultural practice strategies to enhance nutritional value of pak choi and in breeding to develop a pak choi cultivar with improved nutritional properties. Specifically, “Asian” was found to be the most responsive to MeJA among investigated cultivars, with increase in glucosinolates and hydrolysis products that are potentially anticarcinogenic and a reduction in a goitrogen compound.

## Figures and Tables

**Table 1:** GC-MS peak intensity changes in hydrolysis products by MeJA foliar spray treatment in pak choi.

**Cultivar**	**Treatment**	**From Aliphatic Glucosinolates (Peak Intensity)^z^**						
		**3-Butenyl ITC ^y^**	**4-Pentenyl ITC**	**Crambene**	**1-Cyano-3,4-Epithiobutane**	**1-Cyano-4,5-Epithiopentane**	**1-Cyano-2-Hydroxy-3,4-Epithiobutane**	**Goitrin**
		**C_5_H_7_NS**	**C_6_H_9_NS**	**C_5_H_7_NO**	**C_5_H_7_NS**	**C_6_H_9_NS**	**C_5_H_7_NOS**	**C_5_H_7_NOS**
Baby bok choy	Control	68.53a ^x^	8.67a	ND ^w^	104.19a	42.72a	20.47a	45.53a
	MeJA	10.76b	4.60b	18.01	20.49b	18.81b	21.48a	6.02b
Chinese cabbage	Control	868.29a	55.55a	7.70	242.24a	41.77a	10.46a	118.07
	MeJA	35.52b	3.33b	ND	23.85b	3.47b	0.96b	ND
Asian	Control	70.70a	10.82b	ND	27.01b	10.07b	5.42b	41.32a
	MeJA	49.19b	24.52a	24.49	35.04a	33.46a	32.52a	25.16b
Col baby choi	Control	501.80a	26.01a	9.00a	298.02a	39.28a	13.66a	81.26
	MeJA	113.48b	18.55b	6.31b	99.48b	28.84b	4.92b	ND
Pak choi pechay	Control	97.93a	15.37a	7.94a	58.26a	28.34a	15.77a	93.61
	MeJA	19.89b	10.91b	8.12a	11.64b	12.98b	6.55b	ND
		**From Indole Glucosinolates (Peak Intensity)**						
		**1-MI3C**		**1-MI3Carx**		**1-MI3ACN**	**I3CA**	**I3A**
		**C_10_H_11_**		**C_10_H_9_NO_2_**		**C_11_H_10_N_2_O**	**C_9_H_7_NO**	**C_10_H_8_N_2_**
Baby bok choy	Control	26.40b		12.61b		5.13b	1.70	0b
	MeJA	72.16a		117.22a		280.24a	ND	27.02a
Chinese cabbage	Control	29.57a		32.24b		10.94b	4.17	0b
	MeJA	25.17a		97.89a		94.18a	ND	11.33a
Asian	Control	39.98b		32.47b		15.60b	3.44	1.42b
	MeJA	103.66a		129.81a		285.38a	ND	25.29a
Col baby choi	Control	39.11b		40.28b		12.05b	4.82	1.47b
	MeJA	90.46a		86.54a		134.25a	ND	13.88a
Pak choi pechay	Control	32.94b		24.43b		12.51b	ND	1.65b
	MeJA	93.14a		102.37a		208.84a	ND	17.86a

^z^ Data represent peak count (×10^3^) of each compound. ^y^ 3-butenyl ITC and 1-cyano-3,4-epithiobutane from gluconapin; 4-pentenyl ITC and 1-cyano-4,5-epithiopentane from glucobrassicanapin; crambene, goitrin and 1-cyano-2-hydroxy-3,4-epithiobutane from progoitrin; 1-MI3C, 1-MI3Carx and 1-MI3AC, from neoglucobrassicin; I3CA and I3A from glucobrassicin. ^x^ Means were separated by Student’s *t*-test at *p* ≤ 0.05 (*n* = 3). ^w^ ND, not detected.

**Table 2:** Fold change of primary metabolites in MeJA-treated pak choi compared to control pak choi.

Cultivar	Amino Acids			Organic Acids		Sugars and Sugar Derivatives				
	Alanine	Valine	Glutamic Acid	Citric Acid	Cinnamic Acid	Maltose	Fructose	Galactose	*myo*-Inositol	Glycerol
“Baby bok choy”	1.92 *^,z^	3.50 *	0.97	1.80 *	4.75 *	0.87	0.47 *	2.66 *	1.73 *	2.02 *
“Chinese cabbage”	2.00 *	1.53	0.83 *	2.40 *	1.31	1.33	0.67 *	6.87 *	1.03	3.33 *
“Asian”	1.14	2.00 *	2.85 *	1.20 *	2.32 *	0.24 *	0.65 *	0.54 *	2.26 *	2.17 *
“Col baby choi”	0.97	0.87	0.93 *	2.25 *	2.98 *	0.24 *	1.17 *	0.89	1.39 *	0.87
“Pak choi pechay”	2.23 *	9.36 *	2.68 *	1.50 *	3.27 *	0.79 *	0.86	4.75 *	1.85 *	2.68 *
Total change ^y^	1.59	2.76	1.42	1.73	3.06	0.51	0.75	2.40	1.48	2.02
VIP score ^x^	1.59	1.12	1.00	1.64	1.22	1.02	1.36	1.04	1.20	1.39

^x^ Variable importance in projection. ^y^ Total change was calculated as the relative ratio of the total peak intensity of each metabolite in MeJA-treated plants to the total peak intensity of control plants (*n* = 3). ^z^ Values were calculated as the fold change compared to the control group. Asterisk (*) indicates a significant difference of the fold change compared to the control by Student’s *t*-test at *p* ≤ 0.05 based on peak intensity (*n* = 3).

## Supplementary Materials

Supplementary materials can be found at http://www.mdpi.com/1422-0067/18/5/1004/s1.
