# Supplementary material for: Cultivar-Specific Changes in Primary and Secondary Metabolites in Pak Choi (Brassica Rapa, Chinensis Group) by Methyl Jasmonate
Source: Int J Mol Sci. 2017 May 7;18(5):1004. doi: 10.3390/ijms18051004 (PMC5454917; doi:10.3390/ijms18051004)

**Cultivar-Specific Changes in Primary and Secondary Metabolites in Pak Choi (*Brassica rapa* Chinensis Group) by Methyl Jasmonate**

Moo Jung Kim^1^, Yu-Chun Chiu^1^, Na Kyung Kim^2^, Hye Min Park^2^, Choong Hwan Lee^2^, John A. Juvik^3^, Kang-Mo Ku^1*^

**Supplementary Table S1**. Relative gene expression in MeJA-treated pak choi compared to control pak choi.

| Gene class | Number ^z^ | Gene name | Cultivar ^y^ | | | | |
| --- | --- | --- | --- | --- | --- | --- | --- |
|  |  |  | P1 | P2 | P3 | P4 | P5 |
|  |  |  | 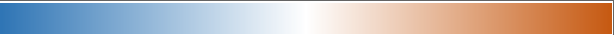 0  1  50 | | | | |
| Chain elongation | 1 | *ELONG* | 0.81 ^x^ | 0.68 | 0.83 | 1.74* | 0.89 |
|  | 2 | *MAM1* | 0.92 | 0.97 | 1.03 | 3.15* | 1.28 |
|  | 3 | *MAM3* | 0.48 | 0.62* | 1.51 | 10.81* | 1.02 |
| Core-structure biosynthesis | 4 | *SUR1* | 0.67* | 1.11 | 0.86 | 1.49* | 0.71 |
|  | 5 | *UGT74B1* | 0.98 | 1.78* | 0.59 | 2.16* | 2.14 |
|  | 6 | *SOT17* | 1.35 | 1.35 | 0.72 | 0.84 | 0.66 |
|  | 7 | *SOT18* | 1.02 | 0.62 | 0.94 | 2.22* | 0.19 |
|  | 8 | *CYP79B2* | 0.38* | 3.77* | 1.66 | 2.98* | 1.56 |
|  | 9 | *SOT16* | 0.73* | 1.69* | 1.72* | 1.35 | 0.61 |
| Secondary modification | 10 | *FMOGSOX2* | 1.00 | 0.81 | 0.94 | 2.36* | 1.44 |
|  | 11 | *AOP2* | 1.13 | 1.51 | 0.92 | 2.32* | 1.57* |
|  | 12 | *OH1* | 15.36* | 0.48* | 49.83* | 0.42* | 7.30* |

^z^Numbers for each gene corresponding to Figure 1.

^y^ P1, “Baby bok choy”; P2, “Chinese cabbage”; P3, “Asian”; P4, “Col baby choi”; P5, “Pak choi pechay”

^x^ Relative gene expression was calculated as the relative ratio of gene expression in
MeJA-treated pak choi to control plants (n = 3). Asterisks (*) indicate a significant difference from the control by Student’s *t*-test at *p* ≤ 0.05.

**Supplementary Table S2**. Glucosinolate profile in control and MeJA-treated pak choi.

| Cultivar | Treatment | Aliphatic glucosinolates (μmol/g DW) | | | | | | | | | | | |
| --- | --- | --- | --- | --- | --- | --- | --- | --- | --- | --- | --- | --- | --- |
|  |  | Glucoiberin | Progoitrin | Glucoalyssin | | Gluconapoleiferin | Gluconapin | | Glucoraphanin | Sinigrin | | Glucobrassicanapin | Glucoerucin |
| “Baby bok choy” | Control | 0.73 ± 0.02 | 1.55 ± 0.08 | 0.25 ± 0.01 | | 0.22 ± 0.01 | 2.45 ± 0.09 | | 0.10 ± 0.00 | 0.13 ± 0.01 | | 2.90 ± 0.13 | ND |
|  | MeJA | 0.06 ± 0.00 | 3.27 ± 0.03* ^y^ | 0.35 ± 0.04 | | 0.84 ± 0.01* | 1.03 ± 0.01* | | 0.12 ± 0.01 | ND | | 1.97 ± 0.03* | ND |
| “Chinese cabbage” | Control | ND ^z^ | 2.30 ± 0.05 | 0.95 ± 0.07 | | 0.18 ± 0.02 | 17.46 ± 0.72 | | ND | ND | | 8.65 ± 0.38 | 0.16 ± 0.01 |
|  | MeJA | ND | 1.11 ± 0.02* | 0.58 ± 0.02* | | ND | 13.92 ± 0.03* | | 0.10 ± 0.00 | ND | | 5.95 ± 0.03* | 0.27 ± 0.01* |
| “Asian” | Control | ND | 1.60 ± 0.02 | 0.31 ± 0.00 | | 0.29 ± 0.00 | 1.32 ± 0.02 | | 0.12 ± 0.01 | ND | | 1.41 ± 0.00 | ND |
|  | MeJA | ND | 5.02 ± 0.16* | 0.92 ± 0.05* | | 1.54 ± 0.04* | 1.57 ± 0.05* | | 0.16 ± 0.00* | ND | | 3.17 ± 0.12* | 0.12 ± 0.00 |
| “Col baby choi” | Control | ND | 1.88 ± 0.06 | 0.33 ± 0.03 | | 0.18 ± 0.02 | 10.60 ± 0.10 | | ND | ND | | 4.88 ± 0.05 | 0.22 ± 0.00 |
|  | MeJA | ND | 0.82 ± 0.06* | 0.31 ± 0.03 | | ND | 9.66 ± 0.46 | | ND | ND | | 6.82 ± 0.34* | 0.36 ± 0.03* |
| “Pak choi pechay” | Control | ND | 2.49 ± 0.17 | 0.22 ± 0.02 | | 0.58 ± 0.08 | 3.02 ± 0.17 | | ND | ND | | 4.45 ± 0.02 | 0.04 ± 0.04 |
|  | MeJA | ND | 2.51 ± 0.07 | 0.22 ± 0.01 | | 1.07 ± 0.02* | 1.61 ± 0.07* | | ND | ND | | 3.71 ± 0.01* | 0.13 ± 0.01 |
| Cultivar | Treatment | Indole glucosinolates (μmol/g DW) | | | | | | | | | | | |
|  |  | Glucobrassicin | | | 4-Hydroxyglucobrassicin | | | 4-Methoxyglucobrassicin | | | Neoglucobrassicin | | |
| “Baby bok choy” | Control | 0.31 ± 0.13 | | | 0.09 ± 0.02 | | | 0.17 ± 0.01 | | | 1.52 ± 0.04 | | |
|  | MeJA | 0.17 ± 0.01 | | | 0.02 ± 0.02 | | | 0.09 ± 0.02* | | | 3.93 ± 0.59* | | |
| “Chinese cabbage” | Control | 0.10 ± 0.05 | | | 0.14 ± 0.03 | | | 0.20 ± 0.03 | | | 1.92 ± 0.21 | | |
|  | MeJA | 0.40 ± 0.10 | | | 0.06 ± 0.01 | | | 0.09 ± 0.01* | | | 5.49 ± 0.33* | | |
| “Asian” | Control | 0.09 ± 0.00 | | | 0.06 ± 0.01 | | | 0.16 ± 0.01 | | | 2.89 ± 0.14 | | |
|  | MeJA | 0.36 ± 0.02* | | | 0.06 ± 0.01 | | | 0.23 ± 0.03 | | | 9.51 ± 0.60* | | |
| “Col baby choi” | Control | 0.19 ± 0.00 | | | 0.12 ± 0.01 | | | 0.32 ± 0.02 | | | 2.74 ± 0.03 | | |
|  | MeJA | 0.44 ± 0.04* | | | 0.09 ± 0.02 | | | 0.31 ± 0.04 | | | 6.93 ± 0.72* | | |
| “Pak choi pechay” | Control | 0.12 ± 0.01 | | | 0.04 ± 0.00 | | | 0.13 ± 0.00 | | | 3.02 ± 0.54 | | |
|  | MeJA | 0.32 ± 0.03* | | | 0.05 ± 0.00 | | | 0.12 ± 0.00 | | | 8.28 ± 0.55* | | |

^z^ ND, not detected.

^y^ Asterisk (*) indicates a significant difference within cultivar by Student’s *t*-test at *p* ≤ 0.05. The concentration of gluconasturtiin was very low (0.1–0.4 µmole/g).

**Supplementary Table S3**. Primary metabolites identified from five pak choi cultivars by GC-TOF-MS analysis.

| Group | Tentative  Metabolites | tR (min)^z^ | Identified ion (*m/z*) | TMS ^y^ | VIP | *p-*value | ID ^x^ |
| --- | --- | --- | --- | --- | --- | --- | --- |
| Amino acids | Alanine | 5.41 | 116 | (TMS)_2_ | 1.59 | <0.001 | STD/MS ^w^ |
|  | Valine | 6.61 | 144 | (TMS)_2_ | 1.12 | <0.001 | STD/MS |
|  | Glycine | 7.51 | 174 | (TMS)_3_ | 0.88 | <0.001 | STD/MS |
|  | Serine | 8.01 | 204 | (TMS)_3_ | 0.62 | <0.001 | STD/MS |
|  | Glutamic acid | 10.18 | 246 | (TMS)_3_ | 1.05 | <0.001 | STD/MS |
|  | Phenylalanine | 10.28 | 218 | (TMS)_2_ | 1.00 | <0.001 | STD/MS |
| Organic acids | Succinic acid | 7.44 | 247 | (TMS)_2_ | 0.64 | <0.001 | STD/MS |
|  | Maleic acid | 7.48 | 245 | (TMS)_2_ | 0.42 | <0.001 | STD/MS |
|  | Fumaric acid | 7.83 | 155 | (TMS)_2_ | 0.57 | <0.001 | STD/MS |
|  | Citric acid | 11.72 | 273 | (TMS)_4_ | 1.64 | <0.001 | STD/MS |
|  | Cinnamic acid | 14.36 | 338 | (TMS)_2_ | 1.22 | <0.001 | MS/MS |
| Sugars and sugar alcohol | Glycerol | 7.19 | 103 | (TMS)_3_ | 1.39 | <0.001 | MS |
|  | Xylose | 10.54 | 103 | (TMS)_4_ | 0.08 | <0.001 | STD/MS |
|  | Adonitol | 11.02 | 217 | (TMS)_5_ | 0.13 | <0.001 | STD/MS |
|  | Galactose | 11.77 | 204 | (TMS)_4_ | 1.04 | <0.001 | MS |
|  | Fructose | 12.16 | 103 | Meox, (TMS)_5_ | 1.36 | <0.001 | STD/MS |
|  | Mannose | 12.53 | 205 | (TMS)_5_ | 0.65 | <0.001 | STD/MS |
|  | *myo*-Inositol | 13.57 | 191 | (TMS)_6_ | 1.20 | <0.001 | STD/MS |
|  | Sucrose | 16.66 | 361 | (TMS)_7_ | 0.90 | <0.001 | STD/MS |
|  | Lactose | 17.16 | 204 | (TMS)_8_ | 0.53 | <0.001 | STD/MS |
|  | Maltose | 17.8 | 361 | (TMS)_8_ | 1.02 | <0.001 | MS |

^z^ Retention time.

^y^ Meox, methyloxime; TMS, trimethylsilyl.

^x^ Identification.

^w^ Metabolites were identified using commercial standard compounds (STD) in comparison with the mass spectra (MS) and retention time.

**Supplementary Table S4**. Identification of glucosinolate hydrolysis products from pak choi.

| Chemical name | Formula  /M.W. | Structure | Mass spectra |
| --- | --- | --- | --- |
| 3-butenyl ITC ^z^ | C_5_H_7_NS  113.18 |  |  |
| 4-pentenyl ITC ^w^ | C_6_H_9_NS  127.21 |  |  |
| Crambene  (1-Cyano-2-hydroxy-3-butene) ^z^ | C_5_H_7_NO  97.12 |  |  |
| 1-Cyano-3,4-epithiobutane ^x,v^ | C_5_H_7_NS  113.18 |  |  |
| 1-Cyano-4,5-epithiopentane ^x,v^ | C_6_H_9_NS  127.21 |  |  |
| 1-Cyano-2-hydroxy-3,4-epithiobutane ^x,v^ | C_5_H_7_NOS  129.18 |  |  |
| Goitrin ^z,x,v^ | C_5_H_7_NOS  129.182 |  |  |
| 1-MI3C ^y^ | C_10_H_11_NO_2_  177.20 |  |  |
| 1-MI3Carx ^x^ | C_10_H_9_NO_2_  175.18 |  |  |
| 1-MI3ACN ^x^ | C_11_H_10_N_2_O  186.21 |  |  |
| I3CA ^x^ | C_9_H_7_NO  145.16 |  |  |
| I3A ^z,x^ | C_10_H_8_N_2_  156.19 |  |  |

^z^ Authentic standard for identification. ^y^ Used the isolated compound for identification [14]. ^x^ Comparison with the NIST library. ^v,w^ Comparison with previous publications ([42,43], respectively).

**Supplementary Table S5**. Primer set information for gene expression analysis.

| Gene class | Gene name | Gene model | Type | Sequence |
| --- | --- | --- | --- | --- |
| Chain elongation | *ELONG* | EF611254 ^z^ | Forward | TTTAGGCTTCGAAGACATCGA |
|  |  |  | Reverse | GTTGATCCCCACCGTGTCCG |
|  | *MAM1* | FJ376038 ^z^ | Forward | CTTAGGCTTCAACGACATCAT |
|  |  |  | Reverse | GTTGATCCCTACCGTGTCCC |
|  | *MAM3* | FJ376040 ^z^ | Forward | CTTAGGCTTCAATGAAATCCA |
|  |  |  | Reverse | GTTGATCCCTACCGTGTCTG |
| Core-structure biosynthesis | *SUR1* | Bol029775 ^y^ | Forward | GCTCCCACGTCCCGTTT |
|  |  |  | Reverse | GCGAACCTCGAGACCACTGT |
|  | *UGT74B1* | Bol005786 ^y^ | Forward | CGACGGCCACGACTTCAT |
|  |  |  | Reverse | GCTTGAAGGATTCGGAGTATGC |
|  | *SOT17* | Bol030757 ^y^ | Forward | CCATCGCCACGCTTCCT |
|  |  |  | Reverse | CCGCCGTACTCGACGAAA |
|  | *SOT18* | Bol026202 ^y^ | Forward | CCCAAAGACAGGCACCACTT |
|  |  |  | Reverse | GGAATCGTCGAAGCGAGATC |
|  | *CYP79B2* | FJ376045 ^z^ | Forward | GTTCTCTGAAAACACTGCAGCG |
|  |  |  | Reverse | TGCTTTTTGTATATCTGATTATCTAC |
|  | *SOT16* | FJ376059 ^z^ | Forward | TTCAAGACGGCAAGAACCAG |
|  |  |  | Reverse | GGGTCAGCAGCTAGCGAG |
| Secondary modification | *FMOGSOX2* | Bol010993 | Forward | CCGGAGCATCTGGATTAATAGC |
|  |  |  | Reverse | CACTTGTTTCTCCCGCTCAAA |
|  | *AOP2* | FJ376073 ^z^ | Forward | GATTGTTCTCGACTCCAAATAGA |
|  |  |  | Reverse | TTTGAATACACGTGGATGCTGC |
|  | *OH1* | FJ376074 ^z^ | Forward | AGGGAGTGATGAAGCTTGCC |
|  |  |  | Reverse | TAAGTCTGGCTCAGGACAGG |
| Endogenous  control gene | *Actin2* | AC189447 ^z^ | Forward | ACGTGGACATCAGGAAGGAC |
|  |  |  | Reverse | CTTGGTGCAAGTGCTGTGAT |

^z^ From Wiesner, Zrenner, Krumbein, Glatt and Schreiner [20].

^y^ From Ku, Becker and Juvik [17].

**Supplementary Figure S1**. Myrosinase activity of control and MeJA-treated pak choi. One unit was defined as 1 μmol of hydrolysis products of glucosinolates released per min.


**Supplementary Figure 2**. GC chromatogram of control (black line) and MeJA-treated (blue line) ‘Baby bok choy’ pak choi after being mixed with horseradish root extract for indirect ESP (epithiospecifier protein) activity measurement by nitrile formation (%). Time offset is used to display two chromatograms.


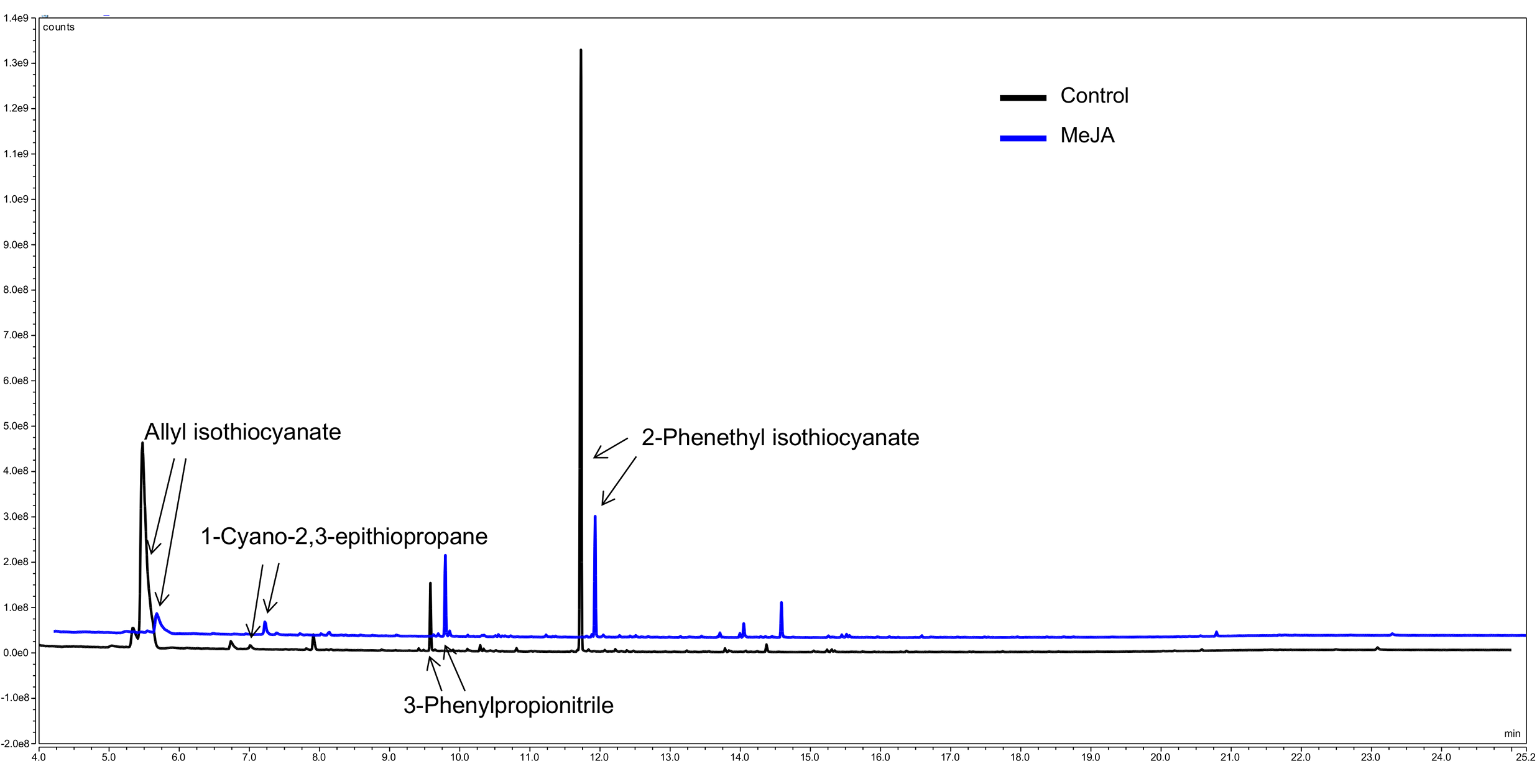

Supplement: Supplementary file 1 [file ijms-18-01004-s001.docx]
